# Understanding alcohol use and changes in drinking habits among people with a severe mental illness: a qualitative framework analysis study

**DOI:** 10.3389/fpsyg.2023.1282086

**Published:** 2023-12-14

**Authors:** Jo-Anne Puddephatt, Harriet Makin, Suzanne H. Gage, Andrew Jones, Laura Goodwin

**Affiliations:** ^1^Spectrum Centre for Mental Health Research, Division of Health Research, Lancaster University, Lancaster, United Kingdom; ^2^Department of Psychology, Edge Hill University, Ormskirk, United Kingdom; ^3^Department of Psychology, Institute of Population Health, University of Liverpool, Liverpool, United Kingdom; ^4^School of Psychology, Faculty of Health, Liverpool John Moores University, Liverpool, United Kingdom

**Keywords:** alcohol, severe mental illness, drinking motives, qualitative, interviews

## Abstract

**Introduction:**

Individuals with a severe mental illness (SMI) are more likely to drink at harmful levels or abstain. While it is known that drinking patterns change over time, the reasons for this among those with a SMI are unclear. This study aimed to (i) explore the experiences with alcohol, particularly in relation to mental health symptoms, and (ii) how drinking patterns have changed over time, among individuals who have a SMI diagnosis, who either currently drink alcohol or no longer drink.

**Methods:**

One-to-one semi-structured telephone interviews were conducted to address the study aims. Current drinkers’ alcohol use was assessed using the Alcohol Use Disorder Identification Test. A framework analysis was used to address the study aims with a specific focus on the differences in the experiences with alcohol use between current and former drinkers.

**Results:**

16 participants were interviewed, and five themes were developed. The analysis highlighted how alcohol was increasingly used to cope with (i) trauma, (ii) SMI-related symptoms, or (iii) stress. Among those with a SMI, non-drinking was facilitated through declines in SMI-related symptoms, previous negative consequences due to alcohol and changing the social environment. Current drinking habits were facilitated through changes in the reasons for drinking and adopting different alcohol moderation techniques.

**Discussion:**

Among those with a SMI diagnosis and who either currently drink alcohol or no longer drink, our findings support the self-medication hypothesis and drinking motives model. However, our findings indicate the need for further development of drinking to cope with a focus on symptoms of a SMI and trauma. Our findings also have implications on specialist alcohol and mental health services, the need to improve individuals’ understanding of SMI, and the need to identify reasons for drinking among those with a recent diagnosis of a SMI.

## 1 Introduction

In 2016, approximately 32.5% of the global population drank alcohol (Griswold et al., 2018), and in 2017, 57.8% of adults in England drank alcohol in the past week (Office for National Statistics, 2018). While 2.2% of adults drank at dependent levels in the past year, globally (Glantz et al., 2020), 3.1% of adults in England drank at dependent levels (levels which can cause health harm; McManus et al., 2016). Alcohol is the leading risk factor for ill-health among those aged 15–49 in England (Public Health England, 2016). Prevalence estimates for different mental health problems vary. In 2019, approximately 301.4 million and 279.6 million people globally had an anxiety or depressive disorder (commonly known as a common mental disorder), respectively (Global Burden of Disease 2019 Mental Disorders Collaborators, 2022). Whereas the prevalence of more severe mental illnesses (SMI, defined as bipolar disorder, schizophrenia or other psychotic condition problems; Hardoon et al., 2013) ranged from 23.6 to 39.5 million depending on the type of problem (Global Burden of Disease 2019 Mental Disorders Collaborators, 2022). Mental health problems are known to contribute towards living with a disability (Whiteford et al., 2015). While the prevalence of a SMI is lower compared with common mental disorders, having a SMI can result in serious functional impairment and interfere with major life activities (National Institute for Mental Health, 2020). It is also known that alcohol and mental health problems often co-occur (Lai et al., 2015; Glantz et al., 2020; Puddephatt et al., 2021), with particularly strong associations between SMIs and harmful alcohol use (Grant et al., 2015; Puddephatt et al., 2021). Given the known associations between alcohol and SMIs, and the burden of having a co-occurring problem, it is important to understand the mechanisms around alcohol use among individuals with a SMI.

The reasons for the associations, and the directionality, between alcohol and SMIs (and mental health) have been debated (Jane-Llopis and Matytsina, 2006; Castillo-Carniglia et al., 2019). More recently, both longitudinal and mendelian randomization research has found more support for declines in mental health and increases in alcohol use (Bell and Britton, 2014; Treur et al., 2021). The increased use of alcohol when experiencing poor mental health may be explained by the self-medication theory whereby people with a mental health problem use alcohol specifically to cope with an acute decline in mental health (Khantzian, 1997). Alcohol may be used initially to enhance low mood or other symptoms but over time this can become a maladaptive coping response and worsen symptoms (Khantzian, 1997). This association can differ across mental health problems. For example, in 2014, 17% of people with bipolar disorder whereas 8% of those with probable psychotic disorder reported harmful drinking (Puddephatt et al., 2021). This suggests that alcohol may be used to cope with specific SMI-related symptoms. For example, among those with bipolar disorder, alcohol consumption increased when experiencing low mood but decreased during the manic phase (Healey et al., 2009). Other research has shown that psychological symptoms associated with their mental health, such as paranoia, reduced the likelihood of using substances as individuals knew it would worsen symptoms (Bradizza and Stasiewicz, 2003). Therefore, coping with SMI-related symptoms may take the form of different drinking patterns (including stopping drinking).

Research has also found significant associations between mental health problems and non-drinking (Puddephatt et al., 2021). This may be because people with poor mental health stop drinking alcohol to manage their mental health, however, the evidence for this is mixed. For example, Ng Fat and colleagues found no significant increases in non-drinking among young people with poor mental health (Ng Fat et al., 2018). Whereas Skogen and colleagues found associations between types of personality disorder and current, but not lifelong, non-drinking (Skogen et al., 2011). It may be that individuals with more severe mental health problems stop drinking due to health reasons (Rosansky and Rosenberg, 2020), symptoms of poor mental health (Bradizza and Stasiewicz, 2003; Healey et al., 2009), being a former harmful drinker or being on medication (Skogen et al., 2011). Due to the severity and implications of having a SMI on the individual, non-drinking may help to manage this diagnosis. A recent study found that 50% of those who met criteria for probable psychotic disorder were non-drinkers (Puddephatt et al., 2021). However, reasons why some individuals with a SMI drink alcohol and others do not are unclear, therefore, it is difficult to understand factors which facilitate changes in drinking patterns among individuals experiencing such mental health problems.

Consequently, the current study aimed to interview individuals with a diagnosis of SMI to explore; (i) the accounts of current and formers drinkers with a SMI on how they used alcohol, (ii) how drinking patterns changed in line with their specific SMI symptoms and, (iii) how their drinking patterns have changed since receiving their diagnosis.

## 2 Materials and methods

Ethical approval was obtained from the Research Ethics Committee at the [University of Liverpool] (ref. 6337). For transparency, the following methods and results are reported in accordance with the COnsolidated criteria for REporting Qualitative research which was developed to improve the quality of reporting for interview and focus group research (Tong et al., 2007; see Supplementary material).

### 2.1 Participants and sample size

Participants were purposively recruited from several community mental health organizations in the North-West of England. Participants were invited to take part in a telephone or online semi-structured interview via a gatekeeper (senior member of staff) from each organization. While the duration of severity of the participant’s SMI was not formally assessed, only those living independently within the community were eligible to take part and the individual’s capacity to participate was further assessed by the gatekeeper of each organization.

The full inclusion and exclusion criteria can be found in Table 1. Briefly, participants were eligible to take part if they (i) were a current drinker or former drinker, (ii) had a self-reported a diagnosis of a SMI and, (iii) lived independently within the community. While not part of the inclusion/exclusion criteria, individuals were eligible to take part if they were currently taking medication given that it was likely that medication would be used to manage their SMI. The university ethics committee were concerned about study participation impacting on recovery for participants with a current or recent AUD diagnosis, specifically whether questions around previous alcohol use would trigger a participant and increase the likelihood of relapse. Therefore, individuals with a self-reported current AUD or previous AUD diagnosis within the last 2 years were excluded. Participants who have had a previous AUD diagnosis or treatment for their alcohol use more than 2 years ago were eligible to take part. Eligibility was confirmed by a trained female postgraduate researcher before arranging a time and date for the interview. While data saturation was not a goal for framework analysis, interviews continued until participants were not providing new information from the data already collected, otherwise known as information redundancy (Saunders et al., 2018).

**Table 1:** Inclusion and exclusion criteria.

Inclusion	Exclusion
Either currently drinks alcohol (defined by self-reporting alcohol use through completing the AUDIT, including those with a previous diagnosis of AUD more than 2 years ago or had an AUDIT score of 16 or higher (indicating harmful drinking)) or is a former drinker (has drank alcohol previously but does not currently drink alcohol) Has a diagnosis of a SMI, including bipolar disorder, schizophrenia, or other psychotic condition (Hardoon et al., 2013; National Health Service England, 2019) Currently lives independently within the community Has received an AUD diagnosis more than 2 years ago	Has a current AUD diagnosis, or received a diagnosis in the last 2 years

### 2.2 Interviews

Seventeen one-to-one semi-structured telephone and online interviews were conducted by a postgraduate researcher (first author), from September to November 2020. Topic guides were developed by the study team and amended using feedback from a participatory involvement group with lived experience of alcohol and mental health problems. The topic guide focused on drinking sessions, symptoms of their SMI, reasons for drinking or not drinking, and support received for their mental health (see Supplementary material). Similar questions were asked in relation to prior to and immediately after receiving their diagnosis (a definitive timepoint for each participant) and their current drinking or non-drinking to allow for comparisons to be made with regards to changes in drinking patterns over time.

### 2.3 Procedure

Each community mental health organization was provided with the inclusion and exclusion criteria and used this to identify potentially eligible participants based on their mental health records. Once potential participants were identified, the gatekeeper contacted individuals about the study. If the individual was interested in taking part and consented to being contacted by the researcher, they were emailed a participant information sheet, outlining the aims and procedure of the study. Participants were prompted to ask questions before agreeing to take part in the study. After participants confirmed that they met inclusion criteria and agreed to take part, were sent a consent form via email to return to the first author. No relationship with participants were established prior to study commencement other than to confirm eligibility and answer questions.

Once a signed consent form was received, the researcher sent all participants a questionnaire to complete ahead of the interview about their demographic information. Current drinkers also completed the Alcohol Use Disorder Identification Test (AUDIT; World Health Organization, 2001) to assess their alcohol use. When the questionnaire was returned, the date and time of interview was arranged. Prior to the interview commencing, participants were required to confirm their name for identification purposes. Interviews were conducted by the first author and audio-recorded using a digital dictaphone with only the researcher present. Upon completion of the interview, participants were debriefed regarding the aims of the study and given details of local and national alcohol and mental health support organizations should they require further support. Participants were also reimbursed with a £20 high-street voucher for their time. Copies of interview transcripts were sent to participants to confirm their accuracy, via email.

### 2.4 Analysis

Data were transcribed verbatim by an external transcriptionist (*n* = 9) or by the first author (*n* = 7). Raw transcripts were checked for accuracy alongside the original audio recordings and then pseudoanonymized based on whether they were a drinker (identified as D) or former drinker (identified as ND). Pseudoanonymized transcripts and responses from the questionnaire were then stored and managed using NVivo 12.

The project team considered multiple qualitative methodologies, including interpretative phenomenological analysis, framework analysis and thematic analysis. Interpretative phenomenological analysis is particularly useful for obtaining a detailed account of an individual’s lived experience and the meaning of a phenomena (Eatough and Smith, 2017) However, as this study aimed to explore experiences with alcohol among a more heterogenous sample (e.g., individuals with different drinking practices) we felt that this approach would not be appropriate. Thematic analysis seeks to explore patterns of shared meanings (Braun et al., 2018). Both thematic and framework analysis are not aligned to a particular epistemological or theoretical approach and explore commonalities and differences within the data. Some of the initial analytical processes are also similar in thematic and framework analysis. A key characteristic of framework analysis is the matrix output where rows are “cases” (e.g., participants) and columns are codes (Gale et al., 2013). Framework analysis allows researchers to develop a matrix and framework based on *a priori* issues and emergent data (Parkinson et al., 2016). Framework analysis is particularly useful for answering four types of research questions; contextual, diagnostic, evaluative, and strategic (Ritchie and Spencer, 1994).

Within the context of this study and the research questions’ focus on (i) individual’s experience with alcohol, (ii) how this relates to symptoms with their SMI diagnosis, and (iii) how their drinking patterns have changed since receiving their diagnosis, we felt that the research questions aligned with contextual and diagnostic research questions from the framework analysis approach. Further, as this study aimed to explore experiences with alcohol among individuals with a SMI who are current drinkers or former drinkers, the framework analysis approach and its matrix allowed for comparisons across cases (including comparisons between current and former drinkers) and within cases. With these considerations in mind, the project team felt that a framework analysis was more appropriate. Framework analysis consists of seven stages; transcription, familiarization, coding, developing a working analytical framework, applying the analytical framework, charting data into the framework matrix, and interpreting the data (Gale et al., 2013).

For the analysis, the first author conducted interviews and made field notes simultaneously. The first author was a British Psychological Society Stage 1 Health Psychology trained postgraduate researcher who had previously completed formal training in conducting qualitative interviews and qualitative analyses and had previously conducted multiple qualitative research studies. The first author familiarized themselves with each transcript and made further reflexive notes and memos about the data. The first author coded two drinker and two non-drinker transcripts using inductive and deductive coding. Some codes were decided *a priori* in accordance with the research questions; however, the majority were developed inductively. An initial framework was developed from this coding and discussed with an experienced postgraduate qualitative researcher who had also read a proportion of transcripts. Amendments were made to the framework and a further four transcripts were coded by the first author. A proportion of transcripts were then randomly selected and second coded by the second author to confirm inter-coder reliability (agreement rate = 83.60%). After the second coding was complete, final changes to the codebook were made. Coding using the framework was then conducted with the remaining transcripts. Coding was facilitated by NVivo 12. Data from Nvivo 12, including AUDIT scores, were then charted into a matrix using Microsoft Excel with summaries of participants provided by category or code. The first author then interpreted data to identify patterns both within the whole sample and subsample of drinkers (including AUDIT scores) and former drinkers to explore similarities and differences between the groups. This was facilitated by interpreting and reviewing similarities or differences between categories and codes in the matrix while reports were also produced in Nvivo 12 to explore these interpretations with reference to raw data from transcripts to further validate potential themes and subthemes. The research group met regularly to discuss potential themes and subthemes before these were finalized.

## 3 Results

Nineteen participants were approached by gatekeepers from community mental health organizations and consented to be contacted by the first author. Two participants could not be reached by the first author. Seventeen participants were eligible and consented to take part. One participant was subsequently excluded as they disclosed in the interview that they have never drank alcohol. This participant was reimbursed for their time. No repeat interviews were conducted. In total, 16 participants (drinkers = 9, former drinkers = 7) took part. The mean duration of interviews was 39 min and 59 s.

### 3.1 Participant characteristics

There were an equal number of males and females, the majority aged between 35 and 54 and of non-White British ethnicity. Among current drinkers, four were low-risk, two hazardous and three harmful drinkers (see Table 2). Two former drinkers disclosed that they received an AUD diagnosis in the past but this occurred more than 2 years ago, therefore, were still eligible to take part. Both received formal alcohol treatment through detoxification though one received treatment for their drinking prior to their SMI diagnosis while the other received treatment for their drinking after their SMI diagnosis.

**Table 2:** Participant characteristics.

	*n*	%
Gender		
Male	8	50.0
Female	8	50.0
Age		
25–34	1	6.3
35–44	6	37.5
45–54	6	37.5
55–64	3	18.8
Marital status		
Single	6	37.5
Married/civil partnership	4	25.0
Divorced/separated/widowed	6	37.5
Ethnicity		
White British	7	43.8
White Other	3	18.8
Asian British	4	25.5
Mixed Other	1	6.3
Other	1	6.3
Education		
University degree or higher	7	43.8
Teaching qualifications, BE, BTEC	2	12.5
A-Levels		
GCSE’s (grade C or above)	3	18.8
GCSE’s (grade D-F)	3	18.8
	1	6.25
Mental health diagnosis		
Bipolar disorder only or with one other mental health diagnosis	8	50.0
Schizophrenia or any other psychotic disorder	8	50.0
AUDIT score (for drinker sample)		
Low-risk drinker (AUDIT score of 0–7)	4	44.4
Hazardous drinker (AUDIT score of 8–15)	2	22.2
Harmful drinker (AUDIT score of 16–19)	3	33.3

### 3.2 Overview of links between themes and subthemes

**Figure 1:**
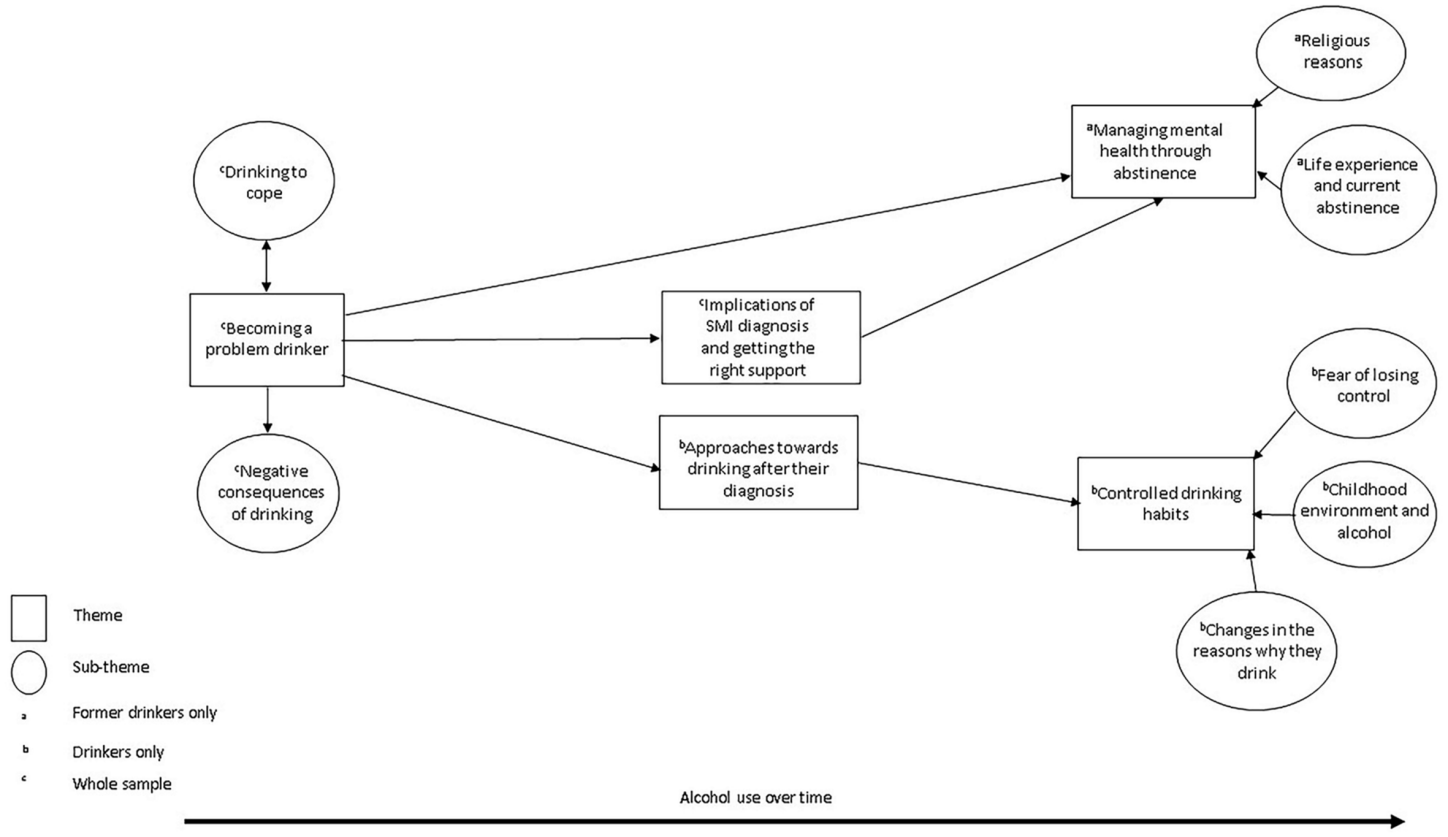
Thematic map.

Five themes were developed and related to experiences with alcohol, how their drinking changed in line with specific SMI symptoms and their perceptions of their current drinking (see Figure 1). The following sections describes each theme and subtheme with reference to supporting quotes. The thematic map shows the process of participants’ changes in drinking, including links between themes and subthemes, which are summarized below.

Regarding the thematic map, “becoming a problem drinker” reflects the increased use of alcohol before participants received support for their mental health, specifically their experiences of using alcohol to cope with trauma, SMI-related symptoms, and significant life events with participants experiencing negative social and physical consequences through their increased drinking. Participants sought help due to either significant declines in their mental health or their drinking had worsened which led to changes in drinking patterns; hence links from “becoming a problem drinker” theme to “implications of SMI diagnosis and getting the right support” themes. Former drinkers described managing their mental health through not drinking alcohol hence the link between themes “becoming a problem drinker” to “managing mental health through abstinence.” Whereas current drinkers experimented with alcohol in different ways before their current drinking habits hence the link between themes “approaches to drinking after diagnosis” and “controlled drinking habits.” Both current and former drinkers’ habits were facilitated by getting mental health or alcohol support which they felt was improving their wellbeing and/or drinking. Current drinking habits were also influenced by receiving their current SMI diagnosis which led to appropriate treatment hence the link between “implications of SMI diagnosis and getting the right support” with “managing mental health through abstinence” and “controlled drinking habits,” respectively.

### 3.3 Theme one – becoming a problem drinker (relates to complete sample)

Prior to their SMI diagnosis, the majority of participants’ initial experiences with alcohol were in social settings. Over time the majority of participants’ drinking increased in quantity and frequency and seemed to be unable to stop drinking once they started. As participants’ drinking increased, alcohol was used more to cope with specific issues or SMI-related symptoms they were experiencing. For participants, alcohol was used to alleviate issues and symptoms but the effects of alcohol were only temporary. Drinking habits became more frequent and heavier, and participants increasingly experienced mental or physical health consequences of their drinking.

*“it kind of just becomes part of your every day, like you know you eat and then you have a drink.” (P2ND)*

There were a minority of participants whose drinking remained steady before their diagnosis. However, for all participants, it was only when their mental health deteriorated and/or drinking had increased that they then sought formal support. This indicates a reluctance to seek support until their mental health and/or drinking had become unmanageable. The impact of their mental health and drinking, and emotions surrounding this, seemed important to participants in seeking formal support with the perception that other routes of managing on their own had been exhausted.

#### 3.3.1 Sub-theme – drinking to cope

Over time, alcohol was used to cope with previous traumatic events (e.g., sexual abuse), symptoms associated with their SMI, or stressful events (e.g., job pressures) which had previously been self-managed without the use of formal support. Both current and former drinkers drank for one or multiple of these reasons which seemed to develop into a cycle as the initial alleviation of remembering events or symptoms was only temporary and felt worse in the days after drinking. This subsequently led to more drinking. This pattern seemed to continue prior to their diagnosis until their mental health had deteriorated to such an extent that they needed to formal support, indicating a need to manage things on their own.

*“I think I was just drinking to mask my depression and mask my thoughts. Whereas if it [alcohol] started to wear away, all I could see was my depression and see it was still there.” (P7D)*

Participants who reported drinking to cope with previous childhood or early adulthood traumatic events, seemed to initially manage this on their own without the use of alcohol but, over time, was used more to cope or forget these experiences. This indicates how processing and managing traumatic incidents without support may become unsustainable over time, and the potential for using other coping techniques to manage these incidents.

*“before my diagnosis erm I probably drank to forget a lot because I suffered a lot of sexual violence…so I erm like would block try and block things out” (P2D)*

Participants who reported drinking to cope with SMI-related symptoms, such as hypomania (e.g., euphoric, very excited), low mood, and hallucinations, experienced these symptoms for several years and initially managed this on their own. Such symptoms worsened over time, and subsequently used alcohol to manage these. However, the patterns of drinking based on the mental health symptoms experienced differed between participants with the same SMI diagnosis. Some participants with bipolar disorder drank more when they felt symptoms of hypomania to calm these feelings down and drank less when feeling depressed but for others with bipolar disorder, it was vice versa. It may be that the severity of these symptoms differed between participants or that alcohol was more effective for different symptoms within different participants.

*“I did when I felt more depressed in that phase the low phase I drank more but when I was in a high state I could kind of do less alcohol” (P4D)*

*“I’d say yes the mid 2000s where I started drinking heavily just to block out the noise you know block out the voices” (P8D)*

Alcohol was also used to cope with on-going events in participants’ personal or work life where it was used to clear thoughts and became their main coping technique for these issues. Using alcohol in this way also seemed to provide support in other aspects of their lives, particularly where they had become more socially isolated because of changes in their personal life.

*“I literally lost everything and then the drinking began in the social housing and I was abusive to people outside. Erm just started to get worse and worse, thinking that my life’s turned upside down…my thoughts were just going, looking forward to that bottle, to that drink, to that erm place that it took me. Erm that solitude. Erm I have not got no friends but I’ve got this drink as a friend.” (P7D)*

#### 3.3.2 Sub-theme - negative consequences of drinking

As participants’ drinking increased, many experienced physical and mental effects from drinking, such as feeling high while drinking but low and sick in the days afterwards. For some participants, they also experienced more negative consequences from their alcohol use over time, such as hospitalisations or getting into trouble with the police. These events seemed to become more apparent and frequent during the later stages of their drinking and shortly before getting support. Such experiences were key in getting formal support because of the implications it was having on both the participant and their family, and the emotions surrounding these events.

*“I kept on getting into trouble with the police and…I felt I wasn’t being heard and just kept on being abusive and had to pay fines. Erm, I got a record of erm you know just being public disorder erm offences and then I suddenly clicked “I do not wanna be that,” “I do not wanna be that horrible person” I wanted to be erm, a proud citizen of myself, for myself and to make my family proud. I seen me erm, me dad’s face when I was in court, and me mum’s face and thought “why am I doing this to them?.” And that’s what really erm helped me to understand where I was going wrong. Like literally ashamed.” (P7D)*

### 3.4 Theme two – implications of SMI diagnosis and getting the right support (relates to complete sample)

There were differences in the experiences of changing of patterns of drinking between former and current drinkers. Former drinkers stopped drinking just before or as they received help for their mental health, and getting the right support helped facilitate this. Whereas current drinkers changed their drinking habits after getting mental health support and their SMI diagnosis. For former drinkers, stopping drinking seemed to be linked with either their mental health support (including the introduction of medication) working better without the use of alcohol, the consequences of their mental health and/or drinking. Some of this may reflect the treatment options for a SMI, where individuals may be likely to be on antipsychotic medication where alcohol use is discouraged due to its interaction with medication. For former drinkers with a previous AUD, they received formal alcohol support, and it was perceived that drinking alcohol was no longer an option.

*“I had a detox and that’s it I have never I have not had a drink since then…I was probably over a year sober when I was diagnosed with my personality disorder” (P1ND)*

*“Since I’ve been on this medication, I’ve been better, I stopped the drinking, my medication’s working better without fighting with the alcohol consumption.” (P5ND)*

For current drinkers, their relationship with alcohol seemed to change while initially engaging with mental health support where alcohol was used less to cope with their SMI-symptoms, and more for enjoyment. Contrary to former drinkers, current drinkers seemed less motivated to stop drinking altogether but this may have been underpinned by their perceived ability to control or moderate their drinking.

*“I’d have a few drinks and it felt like a very different thing it [alcohol] wasn’t so desperate to manage my anxiety it was more because I was just enjoying myself I suppose” (P3D)*

Underpinning this theme were the delays participants experienced in receiving their SMI diagnosis. Some of these delays were due to misdiagnoses or symptoms not deemed severe enough, and were compounded by additional health problems. This theme was also underpinned by the way in which a SMI was perceived by society and the lack of understanding of the implications of the diagnosis. As such, delays and perceptions had implications on receiving appropriate treatment and exacerbated their mental health problems and facilitated drinking to cope.

*“then later that year I tried to seek help and I did actually get seen in [year] by a psychiatrist erm but the problem was I’d just had a couple of surgeries and I was really, really, really down…I was very emotional and crying and even though I did say to my psychiatrist “look the reason why I’m crying is because I’ve waited so long for this appointment [with the psychiatrist] and I’ve just had these operations and I’m really down and in pain at moment but you know there’s a lot going on that I need to talk to you about.” But unfortunately, all she did was just focus on the fact that I was crying ended the appointment and said “you are clearly depressed” and increased my antidepressants. Which then sent me hypomanic again… So and that’s the real shame actually because that’s during the period I’d been drinking and so all it did was send me hypomanic even more and then the drinking just carried on” (P2ND)*

Participants’ experience of receiving an SMI diagnosis seemed to prompt conflicting emotions depending on the specific type of mental health problem. This was partly due to the way it which was perceived by others, for example, personality disorder being less accepted compared with bipolar disorder. For others, alcohol was used to cope with the way in which SMI’s were portrayed.

*“the bipolar was brilliant because that means I could get access to the bipolar medication which you know sort of like settle things down…Err but borderline personality disorder just came out of nowhere erm and I was a bit scared because I did not know what that was. Erm so after that I think it then settled down but it was it was difficult at first but then when we finally got there and then things started to make sense and things started to slot into place and it was it was alright it was ok” (P8D)*

*“the turmoil it [schizophrenia diagnosis] put on to me. Even days when I felt very suicidal, I just thought like erm “am I going to get through this?…” my head was so full of anger, so much pain that er I just did not know what to do so I was drinking a lot.” (P9D)*

The stigma surrounding a SMI and the implications it can have with regards to taking medication seemed to contribute towards a preference for psychological support, but this was often difficult to access. However, participants seemed to benefit from working with mental health professionals to develop a treatment plan which incorporated education around their diagnosis and SMI-related symptoms, and therefore facilitated changes in their drinking habits.

*“…until I found like places where I could do courses for my wellbeing and things like [mental health third sector service] you know… well they referred me actually to [psychiatric service] that’s off [location] and that was like too far to go… so I just said I’ve heard about this place called [mental health third sector service] I said its closer, so he said well you could go there and then that just opened a new chapter up for me” (P1D)*

*“I’ve just had a lot of therapy to deal with like trauma that’s happened to me. So I’m better but it’s not as traumatic anymore and I know that alcohol does not help so I just do not want to do that” (P2D)*

*“I think after I received my diagnosis I became more aware that I had drink that was becoming a problem and it wasn’t it was actually making my mental health condition worse. But you know diagnosis helped me to realise that drink and my diagnosis and medication do not work well together” (P6D)*

This theme reflects the importance of providing additional support to people who have had a recent SMI diagnosis to ensure that they have a good understanding of what their diagnosis means and its implications. This is particularly important as we found that some participants drank to cope with their SMI diagnosis. This theme also highlights the issues participants experienced in getting timely support and one which helped their symptoms. Early identification and appropriate support can have implications on other behaviours, such as alcohol use, hence this theme links with both “managing mental health through abstinence” and “controlled drinking habits.”

### 3.5 Theme three - approaches towards drinking after their diagnosis (relates to drinker sample)

After receiving their diagnosis, current drinkers, as opposed to former drinkers, began to make changes to their drinking habits. Some drinkers stopped drinking completely for a period, others drank less, while others drank more compared to before their diagnosis. During this time, there seemed to be a shift in focus where for some drinkers, managing their mental health through support or medication became a priority, therefore, drinking alcohol was not a viable option.

*“I realised then that I could not mix alcohol and medication together and I did wanna get better in myself and I literally erm stopped it. I went cold turkey” (P7D)*

Other current drinkers, particularly those with harmful AUDIT scores, seemed to drink more after receiving their diagnosis which were exacerbated by issues participants experienced in terms of accepting their SMI diagnosis, and issues with their treatment plan. This highlights the importance of accessing a range of support during the initial stage of diagnosis because of the potential implications it may have on drinking to cope with their diagnosis, hence this theme links with “implications of SMI diagnosis and getting the right support” theme.

*“I was trying to kind of deal with kind of those words you know,…unfortunately having schizophrenia does not mean that er you know the newspapers are very nice to people who are schizophrenia, er schizophrenic you know? …I suppose I drank a bit more to kind of dull that a little bit so that I did not feel as affected by it” (P6D)*

One former drinker stopped drinking after their SMI diagnosis, but this centred around the need for additional mental health support as well as completing formal alcohol treatment to enable the participant to manage their mental health and change their drinking habits. This indicates the need for multiple interventions for those with co-occurring problems.

*“it [the process to change their drinking] was very gradual because the medication was a small amount. So to me the medication and the talking therapies and going to rehab, it’s a lot of intervention really and it took a while for that to sort of clear” (P5ND)*

This theme indicates how initial changes in drinking habits, whether through initial reductions or increases in their alcohol use alongside identifying appropriate support for their mental health, facilitated changes in their relationship with alcohol and the way in which their mental health was managed hence the themes link with “controlled drinking habits” theme.

### 3.6 Theme four - managing mental health through abstinence (relates to former drinker sample)

All former drinkers in this sample stopped drinking completely, without a gradual decrease in their drinking. This change occurred during a significant decline in their mental health or as they received help for their mental health. One former drinker continued to drink initially after their diagnosis due to the need for additional support for their mental health. Former drinkers described the impact drinking had on them, cultural beliefs, and significant life events as key reasons for stopping drinking which were underpinned by appropriate support and adopting new coping techniques to manage their mental health. Some participants self-disclosed a previous AUD diagnosis, therefore, drinking alcohol was no longer an option.

#### 3.6.1 Sub-theme - religious reasons

A minority of former drinkers had previously been affiliated with religious groups which prohibited the consumption of alcohol. However before their diagnosis, they drank to cope with personal issues though this was hidden from their family because of the way in which alcohol was stigmatised. Prior to their diagnosis, some former drinkers were less affiliated with their religion but, after receiving their mental health diagnosis, they seemed to realign with the values of their religion and felt guilty for consuming alcohol.

*“I mean even if it were not for the fact that, you know, religiously I should not be drinking but when I’m hypomanic I’m not really thinking about what I should and should not be doing. So you know I get into relationships and things like that that which I should not be doing if I’m thinking about my religion so that goes out of the window. But at least now [because of my mental health diagnosis] I’m more aware that when I do drink alcohol it does affect me a hell of a lot more and it’s just not a good combination.” (P2ND)*

This sub-theme illustrates the implications of appropriate mental health diagnoses and treatment plans for some religious groups.

#### 3.6.2 Sub-theme – life experience and current abstinence

Among former drinkers, combined with the support that they received, previous experiences with alcohol and managing the social and physical environment were key in maintaining abstinence. However, experiences with maintaining abstinence seemed to differ among former drinkers who had a previous AUD diagnosis and those did not. Former drinkers with a previous AUD diagnosis, experienced severe negative implications because of their drinking, including social isolation and poverty. They described reliving these experiences to maintain abstinence which indicated that while they continued to want to drink alcohol, it was no longer an option.

*“Because if I stop forgetting about the pain the liar will return and I’ll say to myself “it wasn’t that bad I can have a drink”” (P1ND)*

*“when I was at AA and they would preach abstinence completely like you cannot control it, that’s what they were saying you know so. I just wanna touch wood and hope that I do not. It’s almost like I wanna keep away from drama, erm negative people…so that I can not you know trigger, try to avoid the trigger” (P5ND)*

Others used techniques, such as re-creating their social surroundings, to manage abstinence. While drinking was still an option for them, motivations for not drinking seemed underpinned through focusing on their mental health. Therefore, some avoided environments which may invoke desires to drink to manage this. This shows the interplay between the social environment, mental health, and drinking. Participants’ ability to do so seemed to have been sustained through engaging with support over a long period of time. This indicates the importance of time and long-term support on long-term abstinence, particularly where the social environment previously encouraged drinking.

*“I eventually cut contact with the group of friends that I had …I suppose I wasn’t under the influence of them anymore and erm I do not suppose I’m put in a position now when other people around me are drinking except mainly at family occasions” (P8ND)*

### 3.7 Theme five - controlled drinking habits (relates to drinker sample)

Current drinkers, including those with higher AUDIT scores, seemed to control their drinking through stopping drinking before feeling tipsy. Current drinkers also had a better understanding of their drinking habits which was facilitated from the mental health support they had received. Generally, current drinkers were not motivated to stop drinking completely and instead focused on moderating or controlling their alcohol use. This may be an indication of former drinkers previously drinking at levels where it was no longer an option whereas current drinkers may have previously drunk at harmful levels but were able to reduce and moderate their drinking without abstaining.

Participants seemed to use a range of methods in managing this which seemed to stem from initial changes in their drinking shortly after their SMI diagnosis, such as restricting the amount of alcohol and not drinking when they experienced symptoms of their SMI. Previous experiences with heavy drinking, childhood environment, and drinking for different reasons were also used to manage current drinking habits. For those with higher AUDIT scores, alcohol was still used as a last resort to cope where new coping skills may not be effective.

#### 3.7.1 Sub-theme – fear of losing control

The majority of drinkers were fearful of being vulnerable or experiencing negative consequences from drinking which seemed to inform their current approach towards drinking where they restricted to their consumption. This was done through either the amount they consumed during a drinking occasion where they stopped once they felt unable to control their thoughts and behaviours or limited the number of consecutive days they drank. This also seemed to stem from other members of the family having had problems with alcohol.

*“I will not drink two days in a row you know I leave it to one drink once a week because I like feeling in control of things I suppose. Erm I know that I have the disposition same as my parents to use alcohol to manage my, because I’ve got general anxiety disorder as well…” (P3D)*

Given that the underlying sense of fear of losing control did not seem to encourage abstinence, it suggests the importance of motivation and reasons for drinking in the decision to continue drinking after receiving a SMI diagnosis. Nonetheless, this sub-theme highlights the potential role of past experiences on current drinking habits.

#### 3.7.2 Sub-theme – childhood environment and alcohol

As previously mentioned, several drinkers were raised in an environment where their parents had problems with alcohol which seemed to lead to a belief that they were predisposed to drink more problematically. This history seemed to act as a deterrent for continuing to drink at levels of their parents which may be explained by the negative consequences they witnessed through their parent’s drinking.

*“I’ve always erm been afraid of being out of control and if something happened because of what I’ve seen in the past with my dad here when we were growing up and like sick coming down his nose and out of his mouth” (P1D)*

A minority of drinkers in this sample came from a culture where drinking was prohibited and restricted their drinking because they believed they were susceptible to the effects of alcohol due to their lack of exposure to it.

*“I had one erm just so that I could, because I do not really come from a family of drinkers so much. I got a bit worried about my immunity kind of thing to it.” (P6D)*

Despite most drinkers indicated drinking at problematic levels before their diagnosis where they may have had some family history of alcohol problems, it may be that accessing support was key in making them aware of the risks of their drinking, particularly because of their SMI diagnosis.

#### 3.7.3 Sub-theme – changes in the reasons why they drink

Compared to drinking both before and after their SMI diagnosis, current drinkers seemed content with their current drinking which may be indicative of the changes in the reasons why they drink alcohol where it was used for pleasure rather than to cope and had a better understanding of when not to drink alcohol, for example not drinking when they feel anxious. This may have been facilitated by the mental health support they have had.

*“when I’m anxious I know that a drink would calm me down but that is exactly why I do not have one” (P3D)*

However, while participants who drank at harmful levels (as indicated by their AUDIT score) generally changed their reasons for drinking. These participants continued to perceive alcohol as an alternative method of coping with their SMI-related symptoms. This may have been exacerbated by the coronavirus pandemic where some participants experienced difficulties accessing their mental health medication. Though this alternative coping method was no longer the default response to manage their mental health and instead used as a last resort.

*“I had my appointment last month well I’m not going to see him until about Christmas/January time so if that’s [lockdown] going to happen then what the hell am I going to do? Erm you know and because so many other people are, you know, using all the services and stuff, I just think well you need to kind of do a bit of self-medicating sometimes” (P6D)*

## 4 Discussion

The current study explored the accounts of current and former drinkers with a SMI on how they used alcohol, their perceived changes in drinking patterns over time and reasons for drinking and stopping drinking. Among both current drinkers and former drinkers, we found that alcohol was increasingly used to cope before receiving their SMI diagnosis but these reasons were specific to traumatic events, symptoms of their SMI and/or stress that they had experienced. Changes in drinking patterns occurred at different timepoints between former and current drinkers. Most former drinkers stopped drinking either before or while receiving their mental health diagnosis or alcohol problem but for current drinkers this occurred after their mental health diagnosis. We found that former drinkers stopped drinking due to significant deteriorations in their mental health and/or drinking, or engagement with formal mental health and/or alcohol support. Whereas current drinkers changed their drinking habits due to different drinking motivations which were facilitated through the mental health support they received and/or because of events which occurred in the past. We found that harmful drinkers continued to use alcohol as a last resort to manage their SMI-related symptoms. Receiving appropriate support and the implications of receiving a diagnosis of a SMI were important factors for participants and acted as both barriers and facilitators towards managing their mental health and changing their drinking behaviours.

Participants with a SMI drank initially because of the environment they were in, but over time they drank to cope. This supports both the self-medication hypothesis (Khantzian, 1997) and the drinking motives model proposed by Cooper (1994) which posits that individuals drink for either (i) social, (ii) enhancement, (iii) conformity, or (iv) coping reasons. However, our findings extend on these established theories and suggests that drinking to cope was more complex and specific to SMI-related symptoms, trauma, and stressful events, where alcohol only temporarily alleviated these issues and over time was used to manage the return of these problems. While some of these findings are consistent with previous research conducted in the general population, which has found that mental health influenced upcoming changes in heavy drinking (Bell et al., 2015), and that trauma-related drinking to cope mediated the relationship between post-traumatic stress disorder (PTSD) symptoms and alcohol use problems (Hawn et al., 2020). Our findings indicate the need to extend this to other SMI-related symptoms. We found that some participants drank to cope more with symptoms, such as hypomania or low mood, but that drinking patterns differed between participants experiencing similar SMI-related symptoms. For example, some participants with bipolar disorder drank more when they felt low compared to when they felt high but, for others with the same diagnosis, their drinking patterns were reversed. Previous research has found similar findings among those with bipolar disorder (Healey et al., 2009), but we also found that some harmful drinkers continued to use alcohol to cope with their SMI-related symptoms as a last resort. This indicates the need for healthcare professionals to establish drinking patterns in relation to SMI-related symptoms to establish whether additional support is needed.

Changes in drinking patterns between current and former drinkers with a SMI were underpinned by the priorities of the participant and the perceived ability to manage drinking habits. For former drinkers, there was an emphasis on managing their mental health (due to deteriorations in the lead up to their SMI diagnosis and receiving appropriate mental health support) and alcohol was not an option to manage this effectively. Some former drinkers had a previous AUD diagnosis and completed formal alcohol support more than 2 years ago, and felt that drinking was no longer viable which suggests that abstinence was encouraged in formal alcohol treatment (Witkiewitz et al., 2021), even though research indicates that abstinence may not be necessary (Witkiewitz and Tucker, 2020). For current drinkers, their relationship with alcohol changed and was no longer used to cope which was facilitated by the mental health support they received. Current drinkers, including harmful drinkers, felt that they could control or moderate their drinking without this impacting their mental health which may partially explain the differences in the drinking patterns among those with a SMI. Previous research in the general population had found that a prior AUD does not mean that low-risk drinking or abstinence is necessary, however, this study did not consider whether participants had a pre-existing mental health problem (Fan et al., 2019). The differences in the current drinking habits of former and current drinkers who have a SMI diagnosis to that of those in the general population without a SMI may reflect the differences in (i) the priorities of the individual, (ii) the severity of their SMI-related symptoms, and (iii) perceived ability to manage their alcohol use.

In the context of those with a SMI, some research has found benefits of using more specific interventions alongside substance use treatment, such as trauma-focused interventions on PTSD (Roberts et al., 2015), and alcohol use (Roberts et al., 2022). However, much of the previous research has focused on trauma-specific interventions rather than SMI-related symptoms. Therefore, there is a need to (i) understand whether targeting specific SMI-related symptoms changes drinking patterns, and (ii) establish reasons for drinking when assessing a service users alcohol use as this may tailor interventions more appropriately. For example, if someone is drinking to cope with a specific traumatic event (e.g., sexual abuse) then they may require more specialist trauma support in addition to their treatment plan. Whereas is someone is drinking to cope with hallucinations or hypomania then they may require additional support for these symptoms.

Our findings indicate difficulties individuals with a SMI experienced in accessing help for their mental health and alcohol problems which exacerbated their mental health and facilitated their drinking habits to manage this. Research has shown delays in receiving some SMI diagnoses which may delay access to treatment (Patel et al., 2015), and that delays may be longer if measuring when first symptoms of a SMI occurred (Hirschfeld and Vornik, 2003; Chen et al., 2019). In the UK, there is acknowledgement of the need to provide support for individuals with co-occurring alcohol and mental health problems through any route (Public Health England, 2017), but there is limited guidance on alcohol use with the last Alcohol Strategy published in 2012 (Home Office, 2012). Research also indicates that providing support and adherence to treatment may be compounded by individual’s perception of treatment (Jawad et al., 2018). We found that perceptions of support and value or efficacy of medication contributed towards both the increased and sustained changes in drinking behaviour among both current and former drinkers in this sample. Therefore, there is a need to understand how to improve access to services for both assessment and treatment of a SMI while also considering individual perceptions on treatment pathways when diagnosed with a SMI.

We also found that it took time for participants to accept their SMI diagnosis, including the perceptions and implications of this. For some participants, there was a lack of understanding of their SMI diagnosis while for others, alcohol was used to cope with the stigma of having a SMI. Our findings support previous qualitative research among service users with psychosis which found that there was a lack of understanding of psychosis and increased stigma of having such illness (Burke et al., 2016). Notably, findings indicated a negative perception of having a SMI compared with other mental or physical illnesses and previous research has shown associations between a higher level of internalized stigma with treatment adherence (Livingston and Boyd, 2010). In the context of findings from the current study and among current drinkers, the use of patient education following diagnosis of a SMI may be particularly beneficial. Previous qualitative research found that providing information about medication and personal triggers provided a better understanding of how to use coping techniques (Poole et al., 2015). Other research indicates that acceptance and recovery from these issues may be facilitated through peer support which might be particularly useful where access to professional services is delayed (Davidson and Guy, 2012; Ibrahim et al., 2020). Taken together with previous research, there is a need for follow-up support for newly diagnosed individuals with a SMI to provide information about their diagnosis, different treatment options, and assess other behaviours, such as alcohol use, to determine whether other support is needed.

Finally, other factors, such as childhood environment, contributed towards current alcohol use. For drinkers with a SMI, the impact of heavy parental drinking was described as a major reason for the way in which they drink nowadays, though prior to their diagnosis this group indicated drinking heavily. This reflects the potential influence of exposure to alcohol during childhood on later drinking practices. Researchers have argued the need for further exploration of the way in which children understand and are exposed to alcohol in the family home, and how this can influence later drinking behaviours (Jayne and Valentine, 2017). Indeed the current study indicates how participants’ current drinking habits were influenced by their exposure to heavy parental drinking, and the negative consequences of this. Previous research has shown associations between parental drinking and alcohol expectancies in children (Smit et al., 2019), and predicting adolescent/problem drinking (Ryan et al., 2010; Yap et al., 2017). However, there has been less research to understand declines in later alcohol use among individuals who have a SMI with a family history of harmful drinking though one qualitative study among young people noted that some did not drink because of this (Törrönen et al., 2019). Interestingly this was less salient among former drinkers in this study whereby their mental health, previous drinking habits and the consequences of their drinking seemed to inform their current non-drinking habits. This indicates that a reflection of previous drinking habits (whether their own or others) informed current drinking habits among those with a SMI. As such, this may have been facilitated through engagement with mental health organisations, treatment for their SMI, and motivations to drink. However, the role of past drinking behaviours, parental drinking, and engagement with mental health support should be explored further among those with a SMI.

### 4.1 Strengths and limitations

Our study provides a unique insight into two different patterns of drinking among individuals with a SMI and how this changed over time in relation to their SMI diagnosis. Specifically, this study provides a novel insight into both current and former drinkers experience with alcohol. Nonetheless our study is not without limitations. Firstly, we explored alcohol use among individuals with a SMI who lived independently in the community. There may be differences in our findings compared to individuals recruited from secondary care who were not engaged with community mental health organisations or those not currently engaged with mental health support services. We chose to focus on those living independently in the community as we sought to explore patterns among drinkers and former drinkers, whereas including individuals from inpatient secondary services would be prohibited from drinking at the time of interviews taking place. While this research focused on former and current drinkers currently engaged with mental health support, there may have been bias in their experiences with mental health support. Nonetheless, interviews were not focused on their experiences with specific mental health services, rather their experiences in accessing and receiving support more generally. With these issues in mind, there are potential differences in the experiences of alcohol use among former drinkers and current drinkers who were not currently engaged with mental health support which could be explored in future research.

Secondly, our interviews were conducted during the coronavirus pandemic whereby research suggests that there have been changes in drinking patterns and mental health (Irizar et al., 2021; Jacob et al., 2021); we attempted to account for this in the interviews whereby questions around alcohol use during the pandemic were asked to explore potential changes during this time compared to their usual drinking or non-drinking habits. However, this factor did not seem to have a marked impact on their current drinking for most participants. This may have been because most participants had received their SMI diagnosis several years ago, and subsequently had received or were engaged in support.

Thirdly, our findings of changes in drinking and the controlled drinking habits of drinkers may reflect our exclusion of individuals with either a current AUD diagnosis, or one in the last 2 years, however, our sample included those who drank at harmful levels. This exclusion was because of concerns raised by the ethics committee around the impact of the nature of the interview questions on recovery. Nonetheless, our sample included former drinkers who disclosed having had an AUD diagnosis more than 2 years ago who felt that alcohol was no longer an option for them after engaging in formal support. However, it is also important to note that previous AUD diagnoses were self-reported by the participant, and these were not confirmed by case records.

## 5 Conclusion

We found that alcohol use was used to cope with trauma, symptoms specific to having a SMI, and stressful life events, particularly prior to participants receiving their SMI diagnosis. The role of getting appropriate support, stigma of having a SMI, understanding their SMI diagnosis, and parental drinking, contributed towards current drinking habits, including non-drinking. Our findings indicate a need to further explore reasons for drinking to cope among those with a SMI. Further, there is a need for better access to mental health and alcohol support for those with a SMI, and providing better education and understanding of SMI for those who have received an SMI diagnosis.

## Scope

It has been established that alcohol use (including non-drinking) and mental health problems co-occur, with associations strongest among individuals with severe mental illnesses (SMI) but the reasons for this are not well understood. The current study is one of the first to explore the experiences with alcohol and how drinking habits changed over time among individuals with a SMI diagnosis who currently drink or former drinkers.

Using qualitative methods, the current study found some support for the self-medication theory and drinking motives model where alcohol was used to cope before participants received their SMI diagnosis, however, drinking to cope was specific to (i) previous traumatic events, (ii) specific mental health symptoms, and (iii) stress. There were differences in the timepoints when drinking habits changed between current and former drinkers. Our analysis indicated that negative consequences of alcohol use, receiving appropriate mental health and/or alcohol support, and changing participants’ relationship with alcohol were key to making long-term changes to their drinking.

Our findings have implications on specialist mental health and alcohol services and supporting individuals with co-occurring alcohol and mental health problems. The current study enhances our understanding of the self-medication theory and drinking motives model in the context of individuals with a SMI.

## Data availability statement

The datasets presented in this article are not readily available because ethical approval only allows them to be distributed amongst the research team. The analytical and framework matrix will be made available upon request to the corresponding author.

## Ethics statement

The studies involving humans were approved by University of Liverpool ethics committee (ref. 6337). The studies were conducted in accordance with the local legislation and institutional requirements. The participants provided their written informed consent to participate in this study.

## Author contributions

J-AP: Conceptualization, Data curation, Formal analysis, Funding acquisition, Investigation, Methodology, Project administration, Visualization, Writing – original draft, Writing – review & editing. HM: Formal analysis, Investigation, Validation, Writing – review & editing. SG: Funding acquisition, Methodology, Supervision, Validation, Writing – review & editing. AJ: Funding acquisition, Methodology, Supervision, Validation, Writing – review & editing. LG: Conceptualization, Formal analysis, Funding acquisition, Methodology, Supervision, Validation, Writing – review & editing.
