# Supplementary material for: Understanding alcohol use and changes in drinking habits among people with a severe mental illness: a qualitative framework analysis study
Source: Front Psychol. 2023 Dec 14;14:1282086. doi: 10.3389/fpsyg.2023.1282086 (PMC10752932; doi:10.3389/fpsyg.2023.1282086)
Supplement: Supplementary file 2 [file Data_Sheet_2.docx]

**Supplementary materials**

### Interview schedules

**For current drinkers**

Introduction

1. Can you describe when you first started drinking?
   1. Prompt: how old were you? Who was it with?
2. When do you think your mental health started getting worse?
   1. Follow-up: At what age did you receive your diagnosis?
3. To what extent do you feel supported in managing your mental health?
   1. Prompt: Have you got anyone who will listen to your problems?
      1. If they say an individual, follow up with:
         1. Where do you tend to talk to them about your problems?
         2. Are they a drinker?
   2. Follow-up: Are there any activities that help you with your mental health?
   3. NB: This will give some indication of any routes they may have used and I can follow this up at a later stage in the interview

Alcohol use prior to diagnosis

1. Could you describe your drinking prior to receiving your diagnosis?
   1. Prompt: When would you find yourself drinking? Who with?
   2. Prompt: Was there a routine to your drinking e.g. drinking on the weekend?
2. Prior to your diagnosis, were there times when you wouldn’t drink?
   1. Prompt: Can you give an example of when this would be?
3. Prior to your diagnosis, how did you feel when you were drinking?
   1. Prompt: Did you notice any changes in your mood?
4. Prior to your diagnosis, how did you feel in the days after you had been drinking?
   1. Prompt: Did you experience any changes in your mood?

Alcohol use shortly after receiving diagnosis

1. After you initially received your diagnosis, how would you describe your drinking?
   1. Prompt: Did this change at all? If so, how come?
2. After you initially received your diagnosis, did you find any aspects of your life change?
   1. If yes, was there a particular moment when this occurred?
3. After you initially received your diagnosis, were there times when you wouldn’t drink?
   1. If yes, can you give an example of when this would be?
4. After you initially received your diagnosis, how did you feel when you were drinking?
   1. Prompt: Were there any changes compared to before your diagnosis?

Current alcohol use

1. How would you describe your drinking these days?
   1. Follow up: Has this changed since the pandemic?
   2. Follow-up: Can you describe a typical drinking session before the pandemic?
   3. Does this differ depending on what you’re drinking?
   4. If so, in what way?
2. When would you say you find yourself wanting to drink?
   1. Prompt: Is it at a certain time of the day, with certain people?
3. How do you feel when you are drinking?Have you ever thought about not drinking altogether?
   1. Follow-up: Why?
   2. Prompt: Could you describe things that stop you from having a drink?
4. Have you ever purposely restricted your drinking?
   1. Follow-up: If so, in what way?

Concluding question

1. Thank you for answering my questions, was there anything else that you wanted to add about your alcohol use that we may have missed?

**For non-drinkers**

Introduction

1. Can you describe when you started drinking?
   1. Prompt: how old were you? Who was it with?
   2. Can you remember how you felt after your first drinking session?
2. When do you think your mental health started getting worse?
   1. Follow-up: At what age did you receive your diagnosis?
3. To what extent to you feel supported in managing your mental health?
   1. Prompt: Have you got anyone who will listen to your problems?
      1. If they say an individual, follow up with:
         1. Where do you tend to talk to them about your problems?
         2. Are they a drinker?
   2. Follow-up: Are there any activities that help you with your mental health?
   3. NB: This will give some indication of any routes they may have used and I can follow this up at a later stage in the interview
4. When did you stop drinking?
   1. Follow-up: Was this before or after receiving your diagnosis?

Alcohol use prior to diagnosis

1. Could you describe your drinking prior to receiving your diagnosis?
   1. Prompt: When would you find yourself drinking? Who with?
   2. Prompt: Was there a routine to your drinking e.g. drinking on the weekend?
2. Prior to your diagnosis, were there times when you wouldn’t drink?
   1. Prompt: Can you give an example of when this would be?
3. Prior to your diagnosis, how would you feel when you were drinking?
   1. Prompt: Did you notice any changes in your mood?
4. Prior to your diagnosis, how would you feel in the days after you had been drinking?
   1. Prompt: Did you experience any changes in your mood?

Alcohol use shortly after receiving diagnosis

1. After you initially received your diagnosis, how would you describe your drinking?
   1. Prompt: Did this change at all? If so, how come?
2. After you initially received your diagnosis, did you find any aspects of your life change?
   1. If yes, was there a particular moment when this occurred??
3. After you initially received your diagnosis, were there times when you wouldn’t drink?
   1. If yes, can you give an example of when this would be?
4. After you initially received your diagnosis, how did you feel when you were drinking?
   1. Prompt: Were there any changes compared to before your diagnosis?

Current non-drinking habits

1. What prompted you to stop drinking?
   1. Follow-up: How did you go about stopping drinking e.g. gradual decrease or stopped completely?
   2. Follow-up: If not mentioned, did your mental health influence your decision?
2. Do you ever find yourself wanting to drink?
   1. Follow-up: If yes, when do you find this happens?
   2. Follow-up: Has this changed since the pandemic?
3. Could you describe things that help stop you from having a drink?
   1. Prompt: are there certain places where you’re less likely to drink?
4. How would you describe other people’s reactions when you tell them you don’t drink?
   1. Follow-up: Does it differ between different people?

Concluding question

1. Thank you for answering my questions, was there anything else that you wanted to add about your alcohol use that we may have missed?
